# Disparities in the Use of General Somatic Care among Individuals Treated for Severe Mental Disorders and the General Population in France

**DOI:** 10.3390/ijerph17103367

**Published:** 2020-05-12

**Authors:** Coralie Gandré, Magali Coldefy

**Affiliations:** Institut de Recherche et Documentation en Économie de la Santé (IRDES), 75019 Paris, France; coldefy@irdes.fr

**Keywords:** severe mental illnesses, somatic care, healthcare use, health inequities, vulnerable populations

## Abstract

Individuals with severe mental illnesses (SMI) face a striking excess and premature mortality which has been demonstrated in several national contexts. This phenomenon, which constitutes a red-flag indicator of public health inequities, can be hypothesized to result from healthcare access issues which have been insufficiently documented so far. In this context, our objective was to explore patterns of general somatic healthcare use of individuals treated for SMI in comparison to those of the general population in France using national health administrative data and a matched case-control study. Differences in the use of general and specific somatic preventive care services, primary care, routine specialized somatic care and admissions to non-psychiatric hospital departments for somatic causes were described between cases and controls after adjustment on differing clinical needs, socio-economic status, and living environment. Our results show a lower use of general preventive care services and of routine specialized somatic care in the SMI population, despite more frequent comorbidities, and a higher occurrence of avoidable hospitalizations, despite higher contacts with primary care physicians. These findings suggest that the health system fails to address the specific needs of this vulnerable population and support the development of measures aimed at reducing this gap.

## 1. Introduction

Severe mental illnesses (SMI), such as psychotic and bipolar disorders, are a set of disabling and chronic conditions with recurring episodes limiting social skills and functional capacities and strongly interfering with interpersonal relationships [1]. In addition to mental and social disabilities, persons with SMI have a high prevalence of somatic medical conditions [2] and face a particularly striking excess and premature mortality in which suicide plays only a limited role [3]. This phenomenon has been qualified as a “scandal” which transgresses international conventions on the human right to health and healthcare [3] and a red-flag indicator of public health inequities [4]. Large-scale figures on this excess mortality have been available for several decades in several national contexts [3,5] but only very recently in others due to new data linkages opportunities [6,7]. 

To reduce this phenomenon, which does not tend to significantly decrease over time [5,8], the generation of more research evidence is needed and has been advocated for worldwide [9,10]. A combination of factors, such as modifiable risk factors at the individual level (e.g., unhealthy diet or smoking), the adverse consequences of the long-term use of psychotic drugs as well as the effect of mental illness on a person’s capacity to maintain health and social support (withdrawal, fear of stigmatization, isolation), is likely to be involved in the premature mortality of individuals with SMI [11,12,13]. However, contextual or systems-level factors may exacerbate these individual factors. The relative life expectancy of individuals with SMI can be considered to be a proxy measure of the quality of health service provision towards this vulnerable population with significant needs for care that may be missed or undertreated [5]. This leads to the hypothesis that people with SMI face obstacles in their access to somatic care and are less likely to receive adequate treatment for non-psychiatric illnesses. These obstacles should be addressed in the short-term as they represent potential health inequities. The right to good quality, acceptable and accessible health services has been underscored by mental health services users and carers as one of the fundamental issues to be improved [14], and has also been identified as one of the essential conditions to ensure physical and mental health equity for all [15].

To better document healthcare access issues for people with SMI, previous research has notably focused on the receipt of appropriate preventive care services and screening for somatic conditions [16]. However, a coherent picture fails to emerge from the literature, which presents equivocal and sometimes conflicting results regarding the existence of disparities in the use of somatic care for this specific population [16,17,18]. In addition, people with SMI have been neglected in health disparities research and the current evidence base remains sparse. A recent systematic review carried out in the US has notably underscored some limited data on individuals with bipolar disorders; on the use of immunizations; and on the effect of variables which could play a moderating role (such as the socio-economic situation of individuals with SMI) [16]. More data is also needed at a large scale and in contexts where this has not yet been documented as healthcare systems and treatment strategies for patients with SMI differ strongly across countries [19]. This is notably of key necessity in the European region where strong health inequities persist and where progress to reduce them is stalling [15].

In this context, our objective was to explore patterns of somatic healthcare use of individuals treated for SMI in comparison to those of the general population at the national scale in France using nationwide population data and a matched case-control study.

## 2. Materials and Methods 

### 2.1. Conceptual Framework

Our research lies at the crossroads of research on healthcare use and on healthcare disparities. Based on Andersen’s model of health services use, we consider that healthcare use results from the combination of a host of factors, including predisposing factors (in particular socio-demographic characteristics), enabling factors (such as an individual’s living environment) and healthcare needs [20]. Based on previous literature, we also specifically define healthcare disparities as differences in access to and quality of healthcare which are not due to clinical appropriateness and patient needs [21].

### 2.2. Setting and Particularities of the National Context Explored

Our study was carried out in France, where mental healthcare has historically had its own specific territorial organization [22,23]. France is also characterized by a high number of hospitals specialized in psychiatry and a low integration of psychiatric departments within general hospitals, while such integration was advocated in the 1990s to improve the somatic care of individuals with SMI [24]. Their excess mortality has been documented only recently at the national scale. The reduction in their life expectancy was found to reach on average 16 years for men and 13 years for women. These individuals also had higher mortality rates than the French general population, whatever the cause of death considered, and a higher incidence of premature mortality [7]. The reduction of health inequities is high on the national political agenda [25], and a better somatic care for people with mental disorders was one of the key actions underscored in the roadmap for mental health and psychiatry issued by the Ministry in charge of health [26]. It is also part of the priority areas identified by a national consortium of psychiatrists, researchers and patients’ association to improve the quality of life of mental health service users [27]. However, current French national guidelines to improve somatic care in the psychiatric population mention as a foreword that no relevant quantitative data currently exists to provide a full picture of screening and care practice, which does not enable objective identification of issues to be resolved in the field [28]. It can be hypothesized that there are marked access issues for this specific population as previous studies in the French national context have shown that they were particularly significant for disabled individuals and individuals affected by chronic disorders [29,30]. 

In terms of healthcare coverage, which can strongly influence healthcare use, France has a universal public health insurance system. However, coverage is not complete. In particular, a small proportion of inpatient care and doctor visits are not reimbursed by the social health insurance. This residual cost of care at the point of access results from compulsory flat-rate contributions for patients for all hospitalizations or visits to community-based physicians or potential excess fees notably charged by specialist physicians such as experienced psychiatrists or gynecologists. However, France has one of the lowest levels of out-of-pocket payments for patients among high-income countries [31]. Most of the population also subscribes to supplementary private voluntary health insurances to cover these cost-sharing obligations, but not all co-payments are reimbursed by private insurances. Specific measures were implemented for people with low incomes, in particular the possibility to receive free supplementary private health insurance (“*couverture maladie universelle complémentaire*”, CMU-C) or a financial assistance for its purchase (“*aide à l’acquisition d’une complémentaire santé*”, ACS). A long-term illness scheme (LTI) was also created to support patients with chronic long-term disorders including SMI (“*affections de longue durée*”, ALD). Patients in this scheme are exonerated from co-payments of any healthcare, service or drug linked with the treatment of their chronic illness and the follow-up of its main side effects [32,33,34].

### 2.3. Study Design and Main Data Source 

This research relies on a nationwide matched case-control study. The data used for it stem from the French national health data system (“*Système national des données de santé*”, SNDS) which contains all billing records from the social health insurance (SHI) which currently covers almost 100% of the resident population [33]. This database provides comprehensive information on healthcare use in community-based settings and in public and private hospitals as well as individual information on the socio-demographic and medical characteristics of patients [35]. It also includes an annual medical mapping tool (“*cartographie médicalisée*”) which identifies beneficiaries of the main French statutory health insurance scheme who suffer from chronic conditions, including a specific category for mental disorders. Their identification is based on the causes of hospitalizations or inclusion in the LTI scheme for chronic disorders, and the prescription of drugs or medical procedures that are tracers because they are specific to the treatment of certain diseases, over a period extending up to five years [36]. Under French law, our research institute (IRDES) benefits from a permanent access to the SNDS data, which does not require any specific ethical approval or informed consent for accessing this data which is fully anonymized [37].

### 2.4. Study Population

The study population comprised adult patients aged between 18 and 65 which were included in the annual medical mapping tool of the SNDS for 2014. Our population of interest (cases) were patients identified as treated for SMI. We operationally defined SMI as including psychotic (ICD-10 codes F20, F21, F22, F23, F24, F25, F28 and F29) and bipolar disorders (ICD-10 code F31). These disorders were chosen because they are defined in the literature as the most severe disorders [38,39], and because unlike depression they are less likely to be consecutive to the development of a somatic disorder, which could have introduced a bias in our findings. The population used for comparison with our population of interest (controls) was selected within the study population who was not identified as treated for SMI. 

### 2.5. Matching between Cases and Controls

To obtain comparable groups in terms of the most significant predisposing and enabling factors of healthcare use [20], we used an exact matching method to identify three controls per case and obtain a balanced number of matched individuals. The following five matching criteria were used: age, gender, local county (“*département*”) of residence, inclusion or not in the scheme covering healthcare costs for low-income groups (CMU-C) and the quintile of a deprivation index calculated at the patient’s residential zip code [40]. This index, named FDep, was specifically developed for the French context. It was however only available in mainland France and not in overseas territories and was therefore not used as a matching criterion in these territories. This index took into account the median household income, the percentage of high school graduates in the population aged 15 years and older, the percentage of blue-collar workers in the active population and the unemployment rate. Quintiles of this index ranged from least deprived (Q1) to most deprived (Q5). Cases who had missing values for any of the variables used in the matching and cases for whom three exact controls could not be found on such variables represented a minority of all cases and were discarded from the analysis. However, we described their main characteristics so that they could be compared to those of the cases who were matched to controls.

### 2.6. Indicators of Healthcare Use

To obtain a complete picture at the system level of the patterns of use of general somatic care of individuals treated for SMI, we focused on several aspects of care using a set of complementary indicators. They included: (1) the use of general preventive care services (immunization and cancer screening) and of specific prevention targeting the adverse effects of antipsychotic drugs (electrocardiogram, blood test, glucose test and cholesterol test); (2) the use of primary care and routine specialized somatic care (in particular dental, gynecological and ophthalmological care); (3) admissions to non-psychiatric hospital departments for somatic causes with a focus on emergency care and avoidable admissions for causes which should not lead to hospitalizations if they were correctly followed-up in primary care [41,42]. These indicators were selected based on the international literature [38], national guidelines to improve somatic care for individuals with SMI [28] and discussion with health professionals. All indicators were calculated for each individual of the study population as either a binary (use/no use) or a count (number of contacts) variable on a two-year period (2015 to 2016) using the SNDS data.

### 2.7. Analysis

We first tabulated the main characteristics (demographics, socio-economic and clinical status …) of patients identified as treated for SMI and of the population without SMI. We also tabulated these characteristics specifically for matched SMI patients and their controls without SMI, with a more detailed focus on their living environment.

The comparison of the patterns of use of general somatic care between cases and controls was initially carried out by calculating the crude rate or mean of each indicator in both matched groups. The significance of differences between groups was then tested using univariate conditional logistic regressions or univariate generalized estimating equations (GEE) models. 

Second, we carried out multivariable analyses to isolate specific associations between a diagnosis of SMI and healthcare use, taking into account observable differences between cases and controls in addition to those that were considered in the matching. Separate models were fitted for each indicator of healthcare use. For binary indicators, we carried out multivariable logistic regression models with a binomial response distribution, a log link function, and a repeated statement for matched cases and controls. All other indicators were count variables which presented over-dispersion (conditional variances far superior to conditional means). For such indicators, we carried out negative binomial regressions including a repeated statement for matched cases and controls. In addition to a diagnosis or not of SMI, considered explicative variables were selected to further account for patients’ differing socio-economic status, clinical needs and living environment (enabling factors of healthcare needs), after testing for correlation among these variables. Magnitude of associations was measured by adjusted odds ratios (AOR) and their 95% confidence intervals (95% CI), which were obtained by exponential transformation of the estimates.

To further adjust on patients’ socio-economic status in addition to variables considered in the matching, we added an indicator of whether or not each individual of the study population received financial assistance for the purchase of complementary health insurance (ACS). 

Regarding clinical characteristics, we considered the overall health state of included individuals by calculating a synthetic comorbidity index specifically developed for the SNDS data using its annual medical mapping tool. This index was adapted to our study population by not including SMI among comorbidities (modified Expenditure-Related Morbidity Index) [43]. We also considered the total length of stay in inpatient psychiatric care over the two-year study period for each individual included in the matched analysis. This enabled further adjustment based on both the severity of mental disorders and the fact that patients with long hospitalizations are mechanically less likely to seek somatic care in the community. This variable was introduced in the model as a categorical variable with five groups. Thresholds were set based on the distribution of the length of stay in the SMI population with an inpatient psychiatric stay and on national administrative definitions of long-term hospitalizations [44].

Regarding the characteristics of the study population’s living environment, we included an indicator of social fragmentation, adapted from the New Zealand Index of Neighbourhood Social Fragmentation (the NeighFrag index) [45], which aimed at measuring quality of social connections and cohesion on a territory (in particular the sharing of common norms and values and place and people’s attachment). This indicator was built using eight census variables which contributed substantively to a principal component analysis and measured mobility, homeownership, marital status, non-family households, single-person households, school-aged children, immigrants and individuals not living in ordinary households [46]. It ordered territories from the most united to the most socially fragmented. We also included a taxonomy of French local geographical areas which classifies territories based on their healthcare accessibility (for primary and secondary care), overall healthcare needs at the population-level and spatial attractiveness. Detailed methodology of the taxonomy is publicly available elsewhere [47]. Finally, we also introduced an indicator of the degree of urbanicity of territories, the urban area zoning (“*zonage en aires urbaines*”, ZAU), which is based on the influence of urban centers approximated by the geographical repartition of jobs and commuting times (nine urban unit categories) [48]. All three indicators characterizing the living environment of individuals of the study population were introduced at their residential zip code. All the data used for their construction was already aggregated at this geographical level and publicly available. No ethical authorization was therefore required to access it.

Finally, we conducted a sensitivity analysis excluding patients who died during the study period. This additional analysis was only conducted for the comparisons of the two matched populations.

All analyses were performed using SAS EG software version 7.15 HF8 (SAS Institute Inc., SAS Campus Drive, Cary, NC, USA).

## 3. Results

### 3.1. Study Population Characteristics

**Figure 1:**
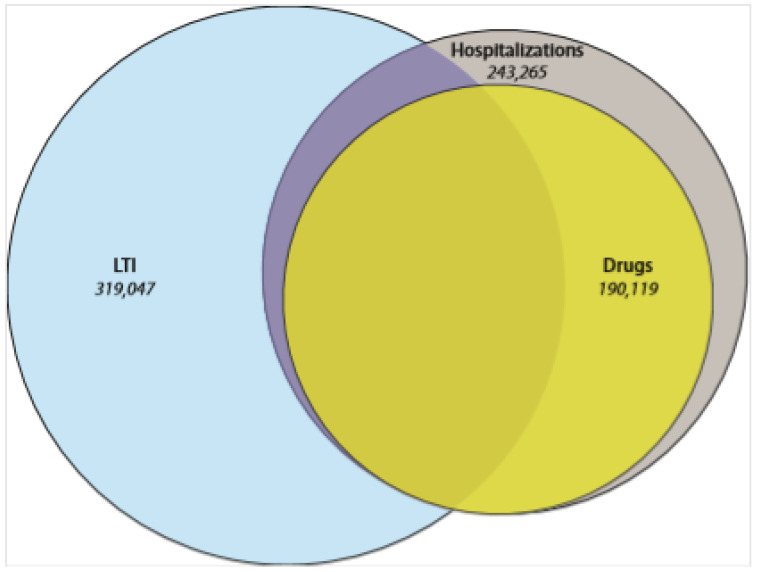
Mode of identification of patients treated for Severe Mental Illnesses (SMI). LTI: Long-Term Illness Scheme.

Overall, 428,093 individuals treated for SMI were identified in France for 2014. Most of these patients (75%) were identified through their inclusion in the LTI scheme for severe mental disorders (concurrently or not to a recent hospitalization for a SMI). The remaining 25% were identified through a hospitalization for a SMI within the last two years, or additionally within the last five years if they also had three deliveries of psychotropic drugs in 2014 [49] (Figure 1). 

Individuals treated for SMI were initially compared to 33,225,644 adult individuals under 65. They were older on average, included more males, and lived in more deprived and socially fragmented territories than the general population. They also presented systematically more frequent comorbidities, except for rheumatoid arthritis or systemic and connective tissue diseases (see Table 1).

Following the matching procedure, 413,437 individuals treated for SMI (97%) were matched with three controls (*n* = 1,240,311). The characteristics of unmatched cases are presented in Table S1. After matching, differences in comorbidities remained between patients treated for SMI and the general population, although they were slightly less marked (Table 2). 

**Figure 2:**
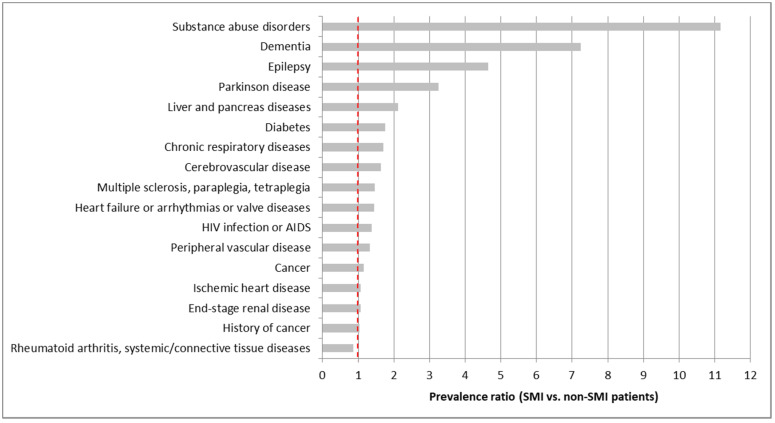
Prevalence ratio of main comorbidities between matched SMI patients and their controls without SMI.

An increased prevalence of comorbidities in the SMI population was in particular observed for comorbid non-SMI mental disorders, neurological disorders, and most other somatic comorbidities. Among the latter, they were particularly significant for liver and pancreas diseases, diabetes, chronic respiratory diseases and cerebrovascular diseases (Figure 2). 

### 3.2. Comparisons of Patterns of Healthcare Use between Matched Cases and Controls

Raw differences in the patterns of healthcare use were observed between matched cases and controls for all indicators of healthcare use. They were systematically significant in the univariate analyses (Table 3). 

Differences remained after adjustment on the study population’s clinical needs, additional socio-economic characteristics, and living environment in the multivariable analyses. 

Regarding use of general preventive care services, a slightly lower use of immunization was observed in the SMI population after adjustment, but the difference was significant only for the hepatitis b vaccine. Differences were more marked for cancer screening. Its use was far less frequent in the SMI population, both for breast and cervical cancer (AOR: 0.68, 95%CI: 0.67–0.69) and colorectal cancer (AOR: 0.81, 95%CI: 0.80–0.82). Specific prevention measures recommended to target the adverse effects of antipsychotic drugs were significantly more frequent in the SMI population but only concerned a limited share of this population.

Regarding the use of primary care, it appeared as slightly more frequent in the SMI population, while on the contrary the average number of contacts with a specialist physician was far lower in this population (AOR: 0.70, 95%CI: 0.70–0.71). All use of routine specialized care was less frequent in the SMI population and differences were particularly strong for gynecological (AOR: 0.63, 95%CI: 0.62–0.64) and ophthalmological care (AOR: 0.71, 95% CI: 0.70–0.72). 

Regarding admissions to non-psychiatric hospital departments, raw differences in the use of emergency care, which was much higher in the SMI population, were strongly reduced in the multivariable analysis. Differences remained far more marked for avoidable hospitalizations (AOR: 2.00, 95% CI: 1.94–2.08). 

**Figure 3:**
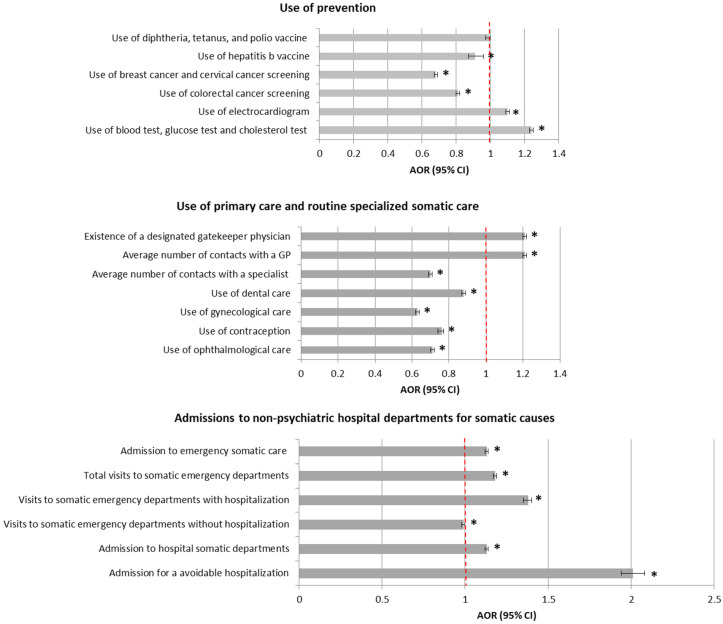
Multivariate analysis of general somatic care use in patients with SMI compared with matched controls without SMI. * Statistically significant.

Associations between all indicators of healthcare use considered and the presence or not of a treated SMI in multivariable analyses are presented in Figure 3, while detailed results of these analyses, including associations with adjustors, are provided in Tables S2–S4. 

Among matched cases and controls, 23,799 (1.4%) died between 2014 and 2016. They represented 3.2% of individuals treated for SMI and 0.9% of controls. When removing such individuals from the comparison of patterns of healthcare use, all our findings on the association between indicators of healthcare use and SMI remained strictly similar, with AOR remaining the same until the two decimal places. 

## 4. Discussion

Individuals treated for SMI in France presented poorer physical health than a matched subset of the general population, with a higher prevalence of comorbid non-SMI mental disorders, neurological disorders, and other somatic comorbidities such as cerebrovascular diseases, diabetes, or liver and pancreas diseases. These multimorbid patients with complex health needs could therefore have been expected to have higher use of somatic care than the general population but our findings demonstrate that the opposite situation was usually observed. Our research demonstrates the sheer scale of the issue as we found quasi systematic associations between the presence of a treated SMI and all indicators of somatic healthcare use even when considering a control population with similar socio-demographics. These differences also remained after adjustment on the study population’s clinical needs, additional socio-economic characteristics and living environment (including both demand and supply-side factors). We notably found a lower use of general preventive care services, in particular cancer screening, and of routine specialized somatic care, despite more frequent comorbidities. We also found a higher occurrence of avoidable hospitalizations in the SMI population, despite higher contacts with primary care physicians.

Our findings, which suggest that individuals treated for SMI constitute an underserved population with unmet somatic healthcare needs, are consistent with those of recent research carried out in the French context. Such research, which was conducted at a lower scale and focused either on the use of hospital care for somatic causes or on the use of routine specialized somatic care, similarly demonstrated disparities between individuals with or without SMI [50,51,52]. The tendency of this vulnerable group to use less routine care (for instance tooth scaling) but more emergency care (for instance dental extractions) [51] was also similar to what we observed using a larger sample and more indicators of healthcare use. International comparisons should be carried out with caution due to possible differences in the assessment of outcomes of interest, in the covariates used in the adjusted analyses or simply in the way SMI populations are identified [16]. With this in mind, our findings presented similarities with research carried out in other national contexts, in particular regarding the lower use of dental care [53] and the increased occurrence of avoidable hospitalizations [54]. However, some inconsistencies were found for the use of primary care. In our study, it was higher for individuals with SMI, as well as in the Netherlands [55], while the contrary was found in New Zealand and in the US [17,56]. The particularity of this indicator of healthcare use should first be noted as it is often not possible to determine whether the primary care contacts were for mental health or somatic issues. Differences with other countries could be explained by the mode of identification of our SMI population. It relied mainly on their inclusion in the LTI scheme for chronic mental disorders which requires an administrative form from a designated gatekeeper physician who is most often a general practitioner (GP). The significant number of contacts between patients with SMI and GPs observed in our study could therefore potentially be driven by visits to obtain this form rather than to receive effective follow-up in primary care as suggested by the increased occurrence of avoidable hospitalizations and the limited use of specialized somatic care in the SMI population. This hypothesis is consistent with previous research which has underscored the overall difficulties of French GPs to address the healthcare needs of individuals with mental disorders [57]. However, at this stage, it is not possible to disentangle to which extent the disparities we objectified resulted from factors at the health professional/system-level (lack of integration of mental and somatic care, misattribution of physical symptoms to mental disorders, complexity of the healthcare system …) or from individual behavioral factors of the SMI population (lack of perception of somatic issues and pain, lack of compliance to treatment, fear of stigma from health professionals, reduced health literacy …). Nevertheless, our findings do suggest that the health system fails to limit ineffective patterns of somatic healthcare use among the SMI population and should focus on addressing their specific needs in the short-term as this can have devastating consequences on their life and life expectancy.

Our study has several strengths. First, we provide an exhaustive picture of the patterns of use of general somatic care of the adult population of individuals treated for SMI at a national scale by using linked claims data covering hospital and community healthcare, which avoids selection and information bias. Relying on objective healthcare consumption data also enables the avoidance of some of the issues faced by research resorting to declarative surveys which are frequently used to study the healthcare use of vulnerable individuals [17,30,58]. These issues include the influence of recall bias and social desirability on the answers provided by respondents as well as specific limits linked to self-report measurement in the population with SMI which may contain biases due to cognition or periodic affective swings [17]. Second, our findings were controlled for differences in clinical, demographic, socio-economic and living environment characteristics of the two populations compared which enabled the isolation of the specific association between SMI and somatic healthcare use taking into account the multiple vulnerabilities faced by individuals with SMI.

Our findings should nevertheless be interpreted in light of limitations which result mainly from the use of health administrative data. Despite its richness, it did not enable us to adjust our analysis on the totality of factors which can be included in the Andersen’s model of health services use [20]. This is notably the case of the subjective perception of one’s own healthcare needs, the attitudes of health professionals towards the SMI population and the subscription or not of a supplementary health insurance scheme (outside of the CMU-C or ACS schemes) and their level of guarantee. This could in particular impact the use of specialists, such as gynecologists or ophthalmologists, for which co-payments are often high due to frequent excess fees charged by these medical specialists. In addition, our analysis was conducted on a population of individuals who received a recent treatment for SMI, and the real prevalence of such disorders is likely to be higher [59]. Our results however provide a conservative estimate as the reduced use of general somatic care is likely to be even higher in a population of SMI individuals who do not receive treatment for their mental disorders. Similarly, our research provides a conservative estimate of disparities in the use of dental care as the data used did not enable us to adjust our analysis on potential dental conditions within the two populations compared while they are likely to be more frequent for individuals with SMI than for the general population [60].

The present study provides opportunities for further research. Based on our first findings, we can hypothesize that even when the SMI population use somatic care, their care is of lesser quality (i.e., less consistent with clinical guidelines) than the one received by the general population. They may for instance benefit from less attention and explanation from physicians, be prescribed different medications for fear of non-compliance, not receive the latest medical innovations… This could be explored, using a similar methodology (exact case-control matching and additional adjustment), by focusing on care pathways for somatic disorders for which there is consensual clinical guidelines and indicators of optimal care such as diabetes or cancer. Research of a qualitative nature could also usefully complement our findings by providing a better understanding of factors at play in the low use of general somatic care by the SMI population, disentangling factors at the patient, professional, and system level and collating patients’ suggestions for improving the current situation.

## 5. Conclusions

Our findings objectify for the first time at the national scale in France the disparities in the use of general somatic care for individuals treated for SMI in comparison to a matched subset of the general population. They suggest that the health system fails to address the specific needs of this vulnerable population and will participate to increase awareness to support the development of measures aimed at reducing these disparities. A holistic approach is notably required for individuals with SMI, putting their needs and experience at the core of the organization and delivery of services in an integrated people-centered approach [61,62]. Accordingly, these measures should be developed in collaboration with SMI patients and their relatives.

## Figures and Tables

**Table 1:** Main characteristics of SMI patients and of the population without SMI.

Characteristics	Patients with SMI (*n* = 428,093)	Population without SMI (*n* = 33,225,644)	Prevalence Ratio
	Mean (±SD) or *n* (%)	Mean (±SD) or *n* (%)	
Demographic characteristics			
Age	45.27 (±11.53)	41.08 (±13.36)	
Sex (female)	198,887 (46.46)	17,976,313 (54.10)	
Socio-economic characteristics at the individual level			
Inclusion in the scheme covering healthcare costs for low-income groups (CMU-C)	56,014 (13.08)	2,990,454 (9.00)	
Missing values	3399 (0.79)	149,753 (0.45)	
Inclusion in the scheme providing financial assistance for the purchase of supplementary health insurance (ACS)	79,953 (18.68)	966,333 (2.91)	
Missing values	3398 (0.79)	149,746 (0.45)	
Characteristics of the living environment			
Quintile of deprivation index (FDep) (from lower to higher deprivation)			
1st quintile	74,727 (17.46)	6,494,616 (19.55)	
2nd quintile	71,097 (16.61)	6,351,714 (19.12)	
3rd quintile	91,747 (21.43)	6,179,252 (18.60)	
4th quintile	82,810 (19.34)	5,983,396 (18.01)	
5th quintile	81,711 (19.09)	6,034,120 (18.16)	
Missing values ^1^	26,001 (6.07)	2,182,546 (6.57)	
Social fragmentation	3.49 (±2.65)	2.38 (±2.73)	
Missing values	18,236 (4.3)	1,733,123 (5.2)	
Residency in an overseas territory	14,000 (3.27)	1,042,582 (3.14)	
Clinical characteristics			
Comorbidity index	1.73 (±3.15)	0.67 (±2.04)	
Ischemic heart disease	7789 (1.82)	443,803 (1.34)	1.36
Cerebrovascular disease	5062 (1.18)	195,192 (0.59)	2.00
Heart failure or arrhythmias or valve diseases	7466 (1.74)	329,593 (0.99)	1.76
Peripheral vascular disease	3312 (0.77)	156,562 (0.47)	1.64
Diabetes	34,432 (8.04)	1,217,620 (3.66)	2.20
Cancer	7139 (1.67)	404,028 (1.22)	1.37
History of cancer	8126 (1.90)	520,134 (1.57)	1.21
Substance abuse disorders	42,423 (9.91)	212,744 (0.64)	15.48
Dementia (including Alzheimer’s disease)	2538 (0.59)	18,038 (0.05)	11.80
Parkinson disease	1685 (0.39)	32,631 (0.10)	3.90
Multiple sclerosis or paraplegia or tetraplegia	2434 (0.57)	115,839 (0.35)	1.63
Epilepsy	10,308 (2.41)	145,005 (0.44)	5.48
Chronic respiratory diseases (including asthma and COPD ^2^)	31,928 (7.46)	1,301,126 (3.92)	1.90
Rheumatoid arthritis or systemic and connective tissue diseases	2139 (0.50)	169,215 (0.51)	0.98
HIV infection or AIDS ^3^	2644 (0.62)	106,911 (0.32)	1.94
End-stage renal disease	617 (0.14)	37,335 (0.11)	1.27
Liver and pancreas diseases (including chronic and acute failures)	10,626 (2.48)	293,445 (0.88)	2.82

^1^ Most missing values were linked to the lack of availability of this index for overseas territories. ^2^ COPD: chronic obstructive pulmonary disease. ^3^ HIV: human immunodeficiency virus; AIDS: acquired immune deficiency syndrome.

**Table 2:** Main characteristics of matched SMI patients and their controls without SMI.

Characteristic	Matched Patients with SMI (*n* = 413,437)	Matched Population without SMI (*n* = 1,240,311)
	Mean (±SD) or *n* (%)	Mean (±SD) or *n* (%)
Demographic characteristics		
Age	45.18 (±11.51)	45.18 (±11.51)
Sex (female)	192,242 (46.50)	576,726 (46.50)
Socio-economic characteristics at the individual level		
Inclusion in the scheme covering healthcare costs for low-income groups (CMU-C)	55,492 (13.42)	166,476 (13.42)
Inclusion in the scheme providing financial assistance for the purchase of supplementary health insurance (ACS)	79,209 (19.16)	42,375 (3.42)
Characteristics of the living environment		
Quintile of deprivation index (FDep) (from lower to higher deprivation)		
1st quintile	47,073 (17.92)	222,219 (17.92)
2nd quintile	70,651 (17.09)	211,953 (17.09)
3rd quintile	91,266 (22.07)	273,798 (22,07)
4th quintile	82,342 (19.92)	247,026 (19.92)
5th quintile	81,244 (19.65)	243,732 (19.65)
Missing values ^1^	13,861 (3.35)	41,583 (3.35)
Social fragmentation	3.48 (±2.65)	2.76 (±2.74)
Missing values	6240 (1.51)	21,660 (1.75)
Residency in an overseas territory	18,864 (3.35)	41,592 (3.35)
Taxonomy of French local geographical areas		
1: Suburban areas with a lower accessibility to healthcare and medium overall health status of the population	54,769 (13.25)	221,705 (17.87)
2: Rural borders with a lower accessibility to healthcare	39,161 (9.47)	140,722 (11.35)
3: Areas with a strong attraction for tourist and retired populations and the best accessibility to healthcare	24,745 (5.99)	86,553 (6.98)
4: Deprived areas, urban and rural, with poor overall health status of the population	41,223 (9.97)	121,311 (9.78)
5: Cities with abundant healthcare supply and heterogeneous socio-economic situations	170,224 (41.17)	415,723 (33.52)
6: Wealthy cities and suburban areas	65,754 (15.90)	203,257 (16.39)
7: Ad hoc category created for overseas territories which present similarities in terms of accessibility to healthcare	13,823 (3.34)	41,166 (3.32)
Missing values	3738 (0.90)	9874 (0.80)
Urban area zoning		
Large urban center	297,030 (71.84)	782,746 (63.11)
Suburban municipality of a large urban center	45,344 (10.97)	196,074 (15.81)
Suburban municipality of several large urban centers	13,077 (3.16)	56,653 (4.57)
Average urban center	13,264 (3.21)	35,914 (2.90)
Suburban municipality of an average urban center	1243 (0.30)	5941 (0.48)
Small urban center	13,453 (3.25)	41,609 (3.35)
Suburban municipality of a small urban center	550 (0.13)	2825 (0.23)
Suburban municipality of several average or small urban centers	12,142 (2.94)	55,365 (4.46)
Isolated municipality located outside the sphere of influence of an urban center	12,036 (2.91)	44,459 (3.58)
Missing values	5298 (1.28)	18,725 (1.51)
Clinical characteristics		
Comorbidity index	1.73 (±3.16)	0.82 (±2.27)
Ischemic heart disease	7528 (1.82)	21,097 (1.70)
Cerebrovascular disease	4883 (1.18)	8992 (0.72)
Heart failure or arrhythmias or valve diseases	7191 (1.74)	14,849 (1.20)
Peripheral vascular disease	3206 (0.78)	7364 (0.59)
Diabetes	33,460 (8.09)	57,041 (4.60)
Cancer	6851 (1.66)	17,855 (1.44)
History of cancer	7817 (1.89)	22,861 (1.84)
Substance abuse disorders	41,066 (9.93)	11,075 (0.89)
Dementia (including Alzheimer’s disease)	2413 (0.58)	933 (0.08)
Parkinson disease	1628 (0.39)	1480 (0.12)
Multiple sclerosis or paraplegia or tetraplegia	2356 (0.57)	4889 (0.39)
Epilepsy	9997 (2.42)	6491 (0.52)
Chronic respiratory diseases (including asthma and COPD ^2^)	31,123 (7.53)	55,126 (4.44)
Rheumatoid arthritis or systemic and connective tissue diseases	2073 (0.50)	7242 (0.58)
HIV infection or AIDS ^3^	2583 (0.62)	5627 (0.45)
End-stage renal disease	601 (0.15)	1741 (0.14)
Liver and pancreas diseases (including chronic and acute failures)	10,313 (2.49)	14,610 (1.18)
Length of stay in inpatient psychiatric care over the two-year study period		
0 days	301,650 (72.96)	1,229,398 (99.12)
1–60 days	65,751 (15.90)	8489 (0.68)
61–180 days	30,299 (7.33)	1882 (0.15)
181–365 days	11,708 (2.83)	417 (0.03)
>365 days	4029 (0.97)	125 (0.01)

^1^ All missing values were linked to the lack of availability of this index for overseas territories. ^2^ COPD: chronic obstructive pulmonary disease. ^3^ HIV: human immunodeficiency virus; AIDS: acquired immune deficiency syndrome.

**Table 3:** Univariate analysis of general somatic care use in patients with SMI compared with matched controls without SMI.

Indicator of Healthcare Use	Matched Patients with SMI (*n* = 413,437) % or Mean (SD)	Matched Population without SMI (*n* = 1,240,311) % or Mean (SD)
Use of prevention *		
Use of general preventive care services		
Use of immunization (diphtheria, tetanus, and polio vaccine) (hepatitis b vaccine)	6.76%	7.07%
Use of immunization (hepatitis b vaccine)	0.78%	0.72%
Use of breast cancer and cervical cancer screening (for women only)	47.93%	60.98%
Use of colorectal cancer screening	6.87%	9.83%
Use of specific prevention targeting the adverse effects of antipsychotic drugs		
Use of electrocardiogram	13.81%	10.36%
Use of blood test, glucose test and cholesterol test (all three)	53.42%	47.58%
Use of primary care and routine specialized somatic care *		
Existence of a designated gatekeeper physician (GP or any other physician)	78.56%	76.45%
Average number of contacts with a GP	13.37 (±15.99)	10.13 (±14.36)
Average number of contacts with a specialist physician ^1^	2.57 (±4.45)	3.77 (±6.82)
Use of dental care	56.74%	61.93%
Use of gynecological care (for women only)	39.96%	53.69%
Use of contraception (for women of child-bearing age only)	38.03%	45.06%
Use of ophthalmological care	34.14%	44.88%
Admissions to non-psychiatric hospital departments for somatic causes *		
Admission to emergency somatic care (in emergency departments)	42.58%	28.10%
Average total number of visits to somatic emergency departments	1.24 (±3.40)	0.54 (±1.41)
Average number of visits to somatic emergency departments followed by a hospitalization	0.32 (±1.13)	0.10 (±0.50)
Average number of visits to somatic emergency departments not precursor to a subsequent hospitalization	0.92 (±2.76)	0.44 (±1.19)
Admission to hospital somatic departments ^2^	34.81%	26.38%
Admission for an avoidable hospitalization ^3^	2.32%	0.76%

* The presence or not of a SMI was significantly associated will all indicators of healthcare use (*p*-value systematically inferior to 0.0001) in the univariate analyses. ^1^ Including cardiologists, dermatologists, gynecologists, gastroenterologists, ophthalmologists, otolaryngologists, and rheumatologists but excluding psychiatrists. ^2^ Excluding hospitalizations in somatic departments for psychiatric conditions or suicide attempts. ^3^ Hospitalizations for asthma, congestive heart failure, chronic obstructive pulmonary disease, dehydration, complications of diabetes, angina (chest pain), dental problem, nutritional deficiency, conditions following immunization.

## Supplementary Materials

The following are available online at https://www.mdpi.com/1660-4601/17/10/3367/s1, Table S1: Characteristics of unmatched SMI patients; Table S2: Use of preventive care services; Table S3: Use of primary care and routine specialized somatic care. Table S4: Admissions to non-psychiatric hospital departments for somatic causes.
