# Supplementary material for: Disparities in the Use of General Somatic Care among Individuals Treated for Severe Mental Disorders and the General Population in France
Source: Int J Environ Res Public Health. 2020 May 12;17(10):3367. doi: 10.3390/ijerph17103367 (PMC7277621; doi:10.3390/ijerph17103367)
Supplement: Supplementary file 1 [file ijerph-17-03367-s001.pdf]

International Journal of Environmental Research and Public Health

MDPI

Supplementary

Table S1. Characteristics of unmatched SMI patients.

Characteristics	Unmatched SMI patients (n=14,656) Mean (±SD) or n (%)
Demographic characteristics	
Age	47,82 (±11.75)
Sex (female)	6,645 (45.34)
Socio-economic characteristics at the individual level	
Inclusion in the scheme covering healthcare costs for low-income groups (CMU-C)	522 (4.64)
Missing values	3,399 (23.19)
Inclusion in the scheme providing financial assistance for the purchase of supplementary health insurance (ACS)	744 (6.61)
Missing values	3,398 (23.19)
Characteristics of the living environment	
Quintile of deprivation index (FDep) (from lower to higher deprivation)	
1st quintile	654 (4.46)
2nd quintile	446 (3.04)
3rd quintile	481 (3.28)
4th quintile	468 (3.19)
5th quintile	467 (3.19)
Missing values[1]	12,140 (82.83)
Social fragmentation	3.99 (±2.60)
Missing values	11,996 (81.85)
Residency in an overseas territory	136 (0.93)
Clinical characteristics	
Comorbidity index	1.55 (3.00)
Ischemic heart disease	261 (1.78)
Cerebrovascular disease	179 (1.22)
Heart failure or arrhythmias or valve diseases	275 (1.88)
Peripheral vascular disease	106 (0.72)
Diabetes	972 (6.63)
Cancer	288 (1.97)
History of cancer	309 (2.11)
Substance abuse disorders	1,357 (9.26)
Dementia (including Alzheimer's disease)	125 (0.85)
Parkinson disease	57 (0.39)

International Journal of Environmental Research and Public Health

Multiple sclerosis or paraplegia or tetraplegia	78 (0.53)
Epilepsy	311 (2.12)
Chronic respiratory diseases (including asthma and COPD[2])	805 (5.49)
Rheumatoid arthritis or systemic and connective tissue diseases	66 (0.45)
HIV infection or AIDS[3]	61 (0.42)
End-stage renal disease	16 (0.11)
Liver and pancreas diseases (including chronic and acute failures)	313 (2.14)
Use of prevention	
Use of general preventive care services	
Use of immunization (diphtheria, tetanus, and polio vaccine / hepatitis b vaccine)	766 (5.23) / 88 (0.60)
Use of breast cancer and cervical cancer screening (for women only)	3,165 (47.63)
Use of colorectal cancer screening	1,216 (8.30)
Use of specific prevention targeting the adverse effects of antipsychotic drugs	
Use of electrocardiogram	1,742 (11.89)
Use of blood test, glucose test and cholesterol test (all three)	6,743 (46.01)
Use of primary care and routine specialized somatic care	
Existence of designated gatekeeper physician (general practitioner or any other physician)	11,567 (78.92)
Average number of contacts with a general practitioner	9.91 (±13.04)
Average number of contacts with a specialist physician[4]	2.40 (±4.29)
Use of dental care	7,356 (50.19)
Use of gynecological care (for women only)	2,330 (35.06)
Use of contraception (for women of child-bearing age only)	536 (32.43)
Use of ophthalmological care	4,759 (32.47)
Admissions to non-psychiatric hospital departments for somatic causes	
Admission to emergency somatic care (in emergency departments)	5,388 (36.76)
Average total number of visits to somatic emergency departments	0.93 (±2.72)
Average number of visits to somatic emergency departments followed by a hospitalization	0.26 (±0.92)

International Journal of
Environmental Research and Public Health

Average number of visits to somatic emergency departments not precursor to a subsequent hospitalization	0.67 (±2.15)
Admission to hospital somatic departments[5]	4,853 (33.11)
Admission for a avoidable hospitalization[6]	362 (2.47)

[1] Most missing values were linked to the lack of availability of this index for overseas territories.

[2] COPD: chronic obstructive pulmonary disease

[3] HIV: human immunodeficiency virus; AIDS: acquired immune deficiency syndrome

[4] Including cardiologists, dermatologists, gynaecologists, gastroenterologists, ophthalmologists, otolaryngologists and rheumatologists but excluding psychiatrists.

[5] Excluding hospitalizations in somatic departments for psychiatric conditions or suicide attempts.

[6] Hospitalizations for asthma, congestive heart failure, chronic obstructive pulmonary disease, dehydration, complications of diabetes, angina (chest pain), dental problem, nutritional deficiency, conditions following immunization.

International Journal of
Environmental Research
and Public Health

MDPI

Detailed results of the multivariable analyses

Table S2. Use of preventive care services.

	Use of immunization (diphtheria, tetanus, and polio vaccine)		Use of immunization (hepatitis b vaccine)		Use of breast cancer and cervical cancer screening (for women only)		Use of colorectal cancer screening		Use of electrocardiogram		Use of blood test, glucose test and cholesterol test (all three)	
**Characteristics**	**AOR (95% CI)**	**Adjusted P-value**	**AOR (95% CI)**	**Adjusted P-value**	**AOR (95% CI)**	**Adjusted P-value**	**AOR (95% CI)**	**Adjusted P-value**	**AOR (95% CI)**	**Adjusted P-value**	**AOR (95% CI)**	**Adjusted P-value**
Clinical characteristics												
Presence of a SMI diagnosis (vs. absence)	0.99 (0.97-1.00)	n.s.	0.91 (0.87-0.96)	0.0002	0.68 (0.67-0.69)	<0.0001	0.81 (0.80-0.82)	<0.0001	1.10 (1.09-1.11)	<0.0001	1.24 (1.23-1.25)	<0.0001
Comorbidity index	0.99 (0.98-0.99)	<0.0001	1.05 (1.05-1.06)	<0.0001	0.98 (0.97-0.98)	<0.0001	1.00 (1.00-1.01)	<0.0001	1.11 (1.10-1.11)	<0.0001	1.06 (1.06-1.06)	<0.0001
Length of stay in inpatient psychiatric care over the two-year study period (ref: >365 days)												
0 days	2.40 (2.00-2.87)	<0.0001	1.55 (1.00-2.38)	0.0477	2.40 (2.14-2.68)	<0.0001	3.78 (3.12-4.59)	<0.0001	0.78 (0.71-0.85)	<0.0001	2.28 (2.14-2.44)	<0.0001
1-60 days	2.47 (2.06-2.96)	<0.0001	2.03 (1.31-3.14)	0.0014	2.07 (1.85-2.32)	<0.0001	2.47 (2.03-3.01)	<0.0001	1.15 (1.06-1.26)	0.0016	2.51 (2.35-2.68)	<0.0001

61-180 days	2.13 (1.77-2.56)	<0.0001	1.65 (1.05-2.57)	0.0285	1.82 (1.62-2.04)	<0.0001	2.17 (1.77-2.64)	<0.0001	1.40 (1.28-1.54)	<0.0001	2.31 (2.16-2.48)	<0.0001
181-365 days	1.59 (1.30-1.94)	<0.0001	1.52 (0.95-2.45)	n.s.	1.32 (1.16-1.50)	<0.0001	1.26 (1.01-1.58)	0.0369	1.24 (1.12-1.37)	<0.0001	1.54 (1.43-1.65)	<0.0001
Socio-economic characteristics at the individual level												
Non-inclusion in the scheme providing financial assistance for the purchase of supplementary health insurance (ACS) (vs. inclusion)	0.98 (0.96-1.01)	n.s.	0.87 (0.82-0.93)	<0.0001	1.34 (1.32-1.37)	<0.0001	1.24 (1.21-1.27)	<0.0001	1.04 (1.02-1.06)	<0.0001	1.02 (1.00-1.03)	0.0101
Characteristics of the living environment												
Social fragmentation	1.00 (0.99-1.00)	0.0149	1.03 (1.02-1.04)	<0.0001	0.96 (0.96-0.97)	<0.0001	0.96 (0.95-0.96)	<0.0001	0.97 (0.97-0.97)	<0.0001	0.97 (0.97-0.97)	<0.0001
Taxonomy of French local geographical areas (ref : *ad hoc* category created for overseas territories which present similarities in terms of accessibility to healthcare)												
1: Suburban areas with a lower	1.12 (1.08-1.17)	<0.0001	0.66 (0.59-0.73)	<0.0001	1.43 (1.39-1.48)	<0.0001	1.55 (1.49-1.62)	<0.0001	0.68 (0.65-0.70)	<0.0001	0.88 (0.86-0.90)	<0.0001

International Journal of *Environmental Research and Public Health* MDPI

accessibility to healthcare and medium overall health status of the population												
2: Rural borders with a lower accessibility to healthcare	1.07 (1.03-1.12)	0.0018	0.64 (0.57-0.73)	<0.0001	1.31 (1.27-1.36)	<0.0001	1.67 (1.60-1.75)	<0.0001	0.62 (0.60-0.64)	<0.0001	0.98 (0.95-1.00)	0.0369
3: Areas with a strong attraction for tourist and retired populations and the best accessibility to healthcare	0.98 (0.94-1.02)	n.s.	0.59 (0.52-0.67)	<0.0001	1.50 (1.45-1.55)	<0.0001	1.40 (1.34-1.47)	<0.0001	0.75 (0.72-0.77)	<0.0001	1.04 (1.01-1.06)	0.002
4: Deprived areas, urban and rural, with poor overall health status of the population	1.15 (1.11-1.2)	<0.0001	0.66 (0.59-0.73)	<0.0001	1.23 (1.19-1.27)	<0.0001	1.48 (1.41-1.54)	<0.0001	0.68 (0.66-0.70)	<0.0001	0.96 (0.94-0.98)	0.0005
5: Cities with abundant healthcare supply and heterogeneous socio-economic situations	1.09 (1.05-1.13)	<0.0001	0.77 (0.70-0.85)	<0.0001	1.38 (1.34-1.42)	<0.0001	1.39 (1.33-1.45)	<0.0001	0.80 (0.78-0.82)	<0.0001	0.92 (0.90-0.94)	<0.0001
6: Wealthy cities and sub-urban areas	1.09 (1.05-1.14)	<0.0001	0.68 (0.62-0.75)	<0.0001	1.55 (1.50-1.59)	<0.0001	1.33 (1.28-1.39)	<0.0001	0.78 (0.76-0.80)	<0.0001	0.88 (0.86-0.89)	<0.0001

International Journal of *Environmental Research and Public Health*

MDPI

Urban area zoning (ref: isolated municipality located outside the sphere of influence of a urban center)												
Large urban center	0.99 (0.95-1.03)	n.s.	0.88 (0.78-0.99)	0.0379	1.11 (1.08-1.14)	<0.0001	0.96 (0.93-0.99)	0.0091	1.03 (1.00-1.07)	n.s.	0.97 (0.95-0.99)	0.0007
Suburban municipality of a large urban center	1.02 (0.98-1.06)	n.s.	0.82 (0.72-0.93)	0.0015	1.10 (1.07-1.13)	<0.0001	0.97 (0.94-1.00)	n.s.	0.96 (0.93-0.99)	0.0218	0.94 (0.93-0.96)	<0.0001
Suburban municipality of several large urban centers	1.06 (1.01-1.11)	0.011	0.82 (0.71-0.95)	0.0094	1.08 (1.04-1.12)	<0.0001	0.98 (0.94-1.02)	n.s.	0.95 (0.91-0.99)	0.0089	0.99 (0.97-1.01)	n.s.
Average urban center	0.96 (0.92-1.01)	n.s.	0.91 (0.79-1.05)	n.s.	1.14 (1.10-1.18)	<0.0001	1.08 (1.03-1.12)	0.0003	0.94 (0.90-0.97)	0.0016	0.93 (0.90-0.95)	<0.0001
Suburban municipality of an average urban center	0.94 (0.85-1.04)	n.s.	0.85 (0.61-1.17)	n.s.	1.12 (1.04-1.21)	0.0025	1.05 (0.97-1.13)	n.s.	0.92 (0.85-1.00)	n.s.	0.90 (0.86-0.95)	<0.0001
Small urban center	1.02 (0.98-1.07)	n.s.	0.85 (0.74-0.99)	0.0327	1.05 (1.02-1.09)	0.0028	1.07 (1.03-1.11)	0.0005	1.03 (0.99-1.07)	n.s.	1.01 (0.98-1.03)	n.s.
Suburban municipality of a small urban center	0.82 (0.71-0.96)	0.0111	0.93 (0.60-1.45)	n.s.	0.95 (0.85-1.05)	n.s.	1.01 (0.91-1.13)	n.s.	0.86 (0.76-0.97)	0.0152	1.00 (0.93-1.07)	n.s.
Suburban municipality of several average or small urban centers	1.04 (0.99-1.08)	n.s.	0.84 (0.73-0.96)	0.0127	1.02 (0.99-1.06)	n.s.	0.93 (0.90-0.96)	<0.0001	0.91 (0.87-0.94)	<0.0001	0.95 (0.92-0.97)	<0.0001

International Journal of Environmental Research and Public Health

MDPI

Table S3. Use of primary care and routine specialized somatic care.

	Existence of a designated gatekeeper physician (GP or any other physician)		Average number of contacts with a GP		Average number of contacts with a specialist physician[1]		Use of dental care		Use of gynecological care (for women only)		Use of contraception (for women of child-bearing age only)		Use of ophthalmological care	
**Characteristics**	**AOR (95% CI)**	**Adjusted P-value**	**AOR (95% CI)**	**Adjusted P-value**	**AOR (95% CI)**	**Adjusted P-value**	**AOR (95% CI)**	**Adjusted P-value**	**AOR (95% CI)**	**Adjusted P-value**	**AOR (95% CI)**	**Adjusted P-value**	**AOR (95% CI)**	**Adjusted P-value**
Clinical characteristics														
Presence of a SMI diagnosis (vs. absence)	1.21 (1.20-1.22)	<0.0001	1.21 (1.20-1.21)	<0.0001	0.70 (0.70-0.71)	<0.0001	0.88 (0.88-0.89)	<0.0001	0.63 (0.62-0.64)	<0.0001	0.76 (0.74-0.77)	<0.0001	0.71 (0.70-0.72)	<0.0001
Comorbidity index	1.06 (1.05-1.06)	<0.0001	1.07 (1.07-1.07)	<0.0001	1.05 (1.04-1.05)	<0.0001	0.96 (0.96-0.96)	<0.0001	0.92 (0.92-0.92)	<0.0001	0.96 (0.96-0.96)	<0.0001	1.01 (1.01-1.01)	<0.0001
Length of stay in inpatient psychiatric care over the two-year study period (ref: >365 days)														
0 days	6.61 (6.19-7.05)	<0.0001	3.48 (3.26-3.71)	<0.0001	2.74 (2.52-2.98)	<0.0001	1.97 (1.84-2.10)	<0.0001	1.69 (1.51-1.89)	<0.0001	1.00 (0.86-1.16)	n.s.	2.57 (2.37-2.79)	<0.0001
1-60 days	6.98 (6.53-7.46)	<0.0001	4.10 (3.84-4.37)	<0.0001	2.41 (2.21-2.62)	<0.0001	2.07 (1.94-2.21)	<0.0001	1.86 (1.65-2.08)	<0.0001	1.13 (0.97-1.31)	n.s.	2.27 (2.09-2.46)	<0.0001

International Journal of Environmental Research and Public Health

61-180 days	5.08 (4.74-5.45)	<0.0001	3.45 (3.22-3.68)	<0.0001	2.10 (1.93-2.29)	<0.0001	1.82 (1.70-1.94)	<0.0001	1.67 (1.48-1.88)	<0.0001	1.10 (0.94-1.29)	n.s.	1.94 (1.79-2.11)	<0.0001
181-365 days	2.43 (2.26-2.62)	<0.0001	2.11 (1.97-2.27)	<0.0001	1.56 (1.42-1.71)	<0.0001	1.28 (1.19-1.37)	<0.0001	1.22 (1.07-1.39)	0.0025	0.99 (0.83-1.18)	n.s.	1.44 (1.31-1.58)	<0.0001
Socio-economic characteristics at the individual level														
Non-inclusion in the scheme providing financial assistance for the purchase of supplementary health insurance (ACS) (vs. inclusion)	1.00 (0.98-1.01)	n.s.	0.87 (0.86-0.88)	<0.0001	1.19 (1.17-1.21)	<0.0001	1.19 (1.17-1.20)	<0.0001	1.39 (1.36-1.41)	<0.0001	0.99 (0.96-1.02)	n.s.	1.31 (1.29-1.32)	<0.0001
Characteristics of the living environment														
Social fragmentation	0.94 (0.93-0.94)	<0.0001	1.00 (1.00-1.00)	<0.0001	0.99 (0.99-0.99)	<0.0001	0.98 (0.98-0.98)	<0.0001	0.98 (0.98-0.99)	<0.0001	0.98 (0.98-0.98)	<0.0001	0.96 (0.96-0.97)	<0.0001
Taxonomy of French local geographical areas (ref : *ad hoc* category created for overseas														

territories which present similarities in terms of accessibility to healthcare)														
1: Suburban areas with a lower accessibility to healthcare and medium overall health status of the population	1.79 (1.74-1.83)	<0.0001	0.94 (0.93-0.95)	<0.0001	1.11 (1.09-1.14)	<0.0001	1.62 (1.58-1.65)	<0.0001	1.27 (1.23-1.32)	<0.0001	1.02 (0.96-1.07)	n.s.	1.32 (1.29-1.35)	<0.0001
2: Rural borders with a lower accessibility to healthcare	1.76 (1.71-1.80)	<0.0001	0.96 (0.95-0.98)	<0.0001	1.03 (1.00-1.05)	n.s.	1.50 (1.47-1.54)	<0.0001	1.08 (1.04-1.12)	0.0001	1.00 (0.94-1.06)	n.s.	1.23 (1.20-1.26)	<0.0001
3: Areas with a strong attraction for tourist and retired populations and the best	1.63 (1.58-1.67)	<0.0001	0.99 (0.97-1.00)	n.s.	1.28 (1.25-1.31)	<0.0001	1.78 (1.74-1.82)	<0.0001	1.28 (1.24-1.33)	<0.0001	0.89 (0.84-0.94)	<0.0001	1.39 (1.36-1.43)	<0.0001

accessibility to healthcare														
4: Deprived areas, urban and rural, with poor overall health status of the population	2.03 (1.98-2.08)	<0.0001	1.08 (1.06-1.09)	<0.0001	1.04 (1.01-1.06)	0.0043	1.38 (1.35-1.41)	<0.0001	1.14 (1.10-1.18)	<0.0001	1.09 (1.03-1.15)	0.0019	1.17 (1.15-1.20)	<0.0001
5: Cities with abundant healthcare supply and heterogeneous socio-economic situations	1.72 (1.68-1.76)	<0.0001	1.02 (1.01-1.03)	0.0069	1.22 (1.19-1.25)	<0.0001	1.60 (1.57-1.64)	<0.0001	1.38 (1.34-1.43)	<0.0001	0.97 (0.92-1.02)	n.s.	1.33 (1.30-1.36)	<0.0001
6: Wealthy cities and sub-urban areas	1.30 (1.27-1.33)	<0.0001	0.87 (0.86-0.88)	<0.0001	1.35 (1.32-1.38)	<0.0001	1.79 (1.76-1.83)	<0.0001	1.68 (1.62-1.74)	<0.0001	0.82 (0.78-0.86)	<0.0001	1.51 (1.48-1.55)	<0.0001
Urban area zoning (ref: isolated municipality located outside the sphere of influence of a urban center)														

International Journal of Environmental Research and Public Health

MDPI

Large urban center	1.05 (1.03-1.08)	<0.0001	1.02 (1.00-1.03)	0.0103	1.15 (1.13-1.17)	<0.0001	1.09 (1.07-1.11)	<0.0001	1.29 (1.25-1.33)	<0.0001	0.96 (0.92-1.01)	n.s.	1.14 (1.12-1.17)	<0.0001
Suburban municipality of a large urban center	1.04 (1.02-1.07)	0.0017	0.99 (0.98-1.00)	n.s.	1.08 (1.06-1.10)	<0.0001	1.09 (1.06-1.11)	<0.0001	1.27 (1.23-1.31)	<0.0001	0.99 (0.94-1.04)	n.s.	1.10 (1.08-1.13)	<0.0001
Suburban municipality of several large urban centers	1.07 (1.04-1.11)	<0.0001	1.02 (1.01-1.04)	0.0056	1.08 (1.06-1.10)	<0.0001	1.07 (1.04-1.09)	<0.0001	1.17 (1.13-1.21)	<0.0001	0.99 (0.93-1.05)	n.s.	1.07 (1.05-1.10)	<0.0001
Average urban center	1.13 (1.10-1.16)	<0.0001	0.97 (0.96-0.99)	0.0005	1.06 (1.03-1.09)	<0.0001	1.09 (1.07-1.12)	<0.0001	1.07 (1.03-1.11)	0.0002	1.00 (0.94-1.06)	n.s.	1.08 (1.06-1.11)	<0.0001
Suburban municipality of an average urban center	1.02 (0.96-1.09)	n.s.	0.86 (0.84-0.89)	<0.0001	0.99 (0.94-1.03)	n.s.	1.08 (1.02-1.13)	0.0048	1.07 (0.99-1.15)	n.s.	1.01 (0.89-1.14)	n.s.	1.05 (1.00-1.10)	n.s.
Small urban center	1.09 (1.06-1.13)	<0.0001	1.02 (1.00-1.03)	0.017	1.05 (1.03-1.08)	<0.0001	1.01 (0.98-1.03)	n.s.	1.09 (1.05-1.13)	<0.0001	1.02 (0.96-1.08)	n.s.	1.07 (1.05-1.10)	<0.0001
Suburban municipality of a small urban center	1.03 (0.94-1.12)	n.s.	0.97 (0.92-1.01)	n.s.	1.04 (0.98-1.11)	n.s.	1.03 (0.96-1.11)	n.s.	1.08 (0.98-1.20)	n.s.	1.08 (0.90-1.30)	n.s.	0.95 (0.88-1.02)	n.s.
Suburban municipality of several average	1.00 (0.98-1.03)	n.s.	0.98 (0.96-0.99)	0.0005	1.03 (1.01-1.06)	0.0011	1.00 (0.97-1.02)	n.s.	1.08 (1.05-1.12)	<0.0001	1.00 (0.95-1.06)	n.s.	1.05 (1.03-1.07)	<0.0001

International Journal of *Environmental Research and Public Health*

MDPI

or small urban centers		

[1] Including cardiologists, dermatologists, gynaecologists, gastroenterologists, ophthalmologists, otolaryngologists and rheumatologists but excluding psychiatrists.

Table S4. Admissions to non-psychiatric hospital departments for somatic causes.

	**Admission to emergency somatic care (in emergency departments)**		**Average total number of visits to somatic emergency departments**		**Average number of visits to somatic emergency departments followed by a hospitalization**		**Average number of visits to somatic emergency departments not precursor to a subsequent hospitalization**		**Admission to hospital somatic departments[1]**		**Admission for a avoidable hospitalization[2]**	
**Characteristics**	**AOR (95% CI)**	**Adjusted P-value**	**AOR (95% CI)**	**Adjusted P-value**	**AOR (95% CI)**	**Adjusted P-value**	**AOR (95% CI)**	**Adjusted P-value**	**AOR (95% CI)**	**Adjusted P-value**	**AOR (95% CI)**	**Adjusted P-value**
Clinical characteristics												
Presence of a SMI diagnosis (vs. absence)	1.13 (1.12-1.14)	<0.0001	1.18 (1.17-1.19)	<0.0001	1.38 (1.35-1.40)	<0.0001	1.14 (1.13-1.15)	<0.0001	0.99 (0.98-1.00)	0.0062	2.01 (1.94-2.08)	<0.0001
Comorbidity index	1.07 (1.07-1.07)	<0.0001	1.08 (1.08-1.08)	<0.0001	1.17 (1.17-1.18)	<0.0001	1.05 (1.05-1.05)	<0.0001	1.16 (1.16-1.16)	<0.0001	1.16 (1.15-1.16)	<0.0001
Length of stay in inpatient psychiatric care over the two-year study period (ref: >365 days)												
0 days	0.33 (0.31-0.35)	<0.0001	0.26 (0.24-0.28)	<0.0001	0.18 (0.17-0.20)	<0.0001	0.29 (0.26-0.31)	<0.0001	0.41 (0.38-0.43)	<0.0001	0.31 (0.27-0.36)	<0.0001

International Journal of Environmental Research and Public Health

MDPI

1-60 days	1.59 (1.49-1.70)	<0.0001	0.86 (0.81-0.93)	<0.0001	0.80 (0.74-0.86)	<0.0001	0.89 (0.82-0.96)	0.0041	1.04 (0.98-1.11)	n.s.	0.48 (0.42-0.55)	<0.0001
61-180 days	1.8 (1.69-1.93)	<0.0001	1.09 (1.02-1.17)	0.0173	1.06 (0.98-1.14)	n.s.	1.10 (1.02-1.20)	0.0196	1.20 (1.12-1.29)	<0.0001	0.56 (0.49-0.65)	<0.0001
181-365 days	1.45 (1.35-1.56)	<0.0001	1.16 (1.07-1.25)	0.0002	1.13 (1.04-1.22)	0.0038	1.17 (1.07-1.28)	0.0008	1.22 (1.13-1.31)	<0.0001	0.77 (0.66-0.90)	0.0013
Socio-economic characteristics at the individual level												
Non-inclusion in the scheme providing financial assistance for the purchase of supplementary health insurance (ACS) (vs. inclusion)	0.85 (0.84-0.86)	<0.0001	0.81 (0.80-0.82)	<0.0001	0.83 (0.82-0.85)	<0.0001	0.80 (0.79-0.82)	<0.0001	0.97 (0.96-0.99)	0.0001	0.72 (0.69-0.75)	<0.0001
Characteristics of the living environment												
Social fragmentation	1.00 (1.00-1.00)	0.016	1.01 (1.01-1.01)	<0.0001	1.00 (1.00-1.01)	0.0022	1.01 (1.01-1.01)	<0.0001	0.99 (0.99-0.99)	<0.0001	1.01 (1.00-1.01)	n.s.
Taxonomy of French local geographical areas (ref : *ad hoc* category created for overseas territories which present similarities in terms of accessibility to healthcare)												

International Journal of Environmental Research and Public Health

1: Suburban areas with a lower accessibility to healthcare and medium overall health status of the population	1.35 (1.32-1.38)	<0.0001	1.23 (1.20-1.27)	<0.0001	0.93 (0.89-0.98)	0.0033	1.32 (1.29-1.36)	<0.0001	1.33 (1.30-1.37)	<0.0001	1.31 (1.18-1.46)	<0.0001
2: Rural borders with a lower accessibility to healthcare	1.45 (1.41-1.48)	<0.0001	1.32 (1.28-1.36)	<0.0001	1.02 (0.97-1.08)	n.s.	1.40 (1.36-1.45)	<0.0001	1.39 (1.36-1.43)	<0.0001	1.42 (1.27-1.58)	<0.0001
3: Areas with a strong attraction for tourist and retired populations and the best accessibility to healthcare	1.25 (1.21-1.28)	<0.0001	1.16 (1.13-1.20)	<0.0001	0.88 (0.83-0.92)	<0.0001	1.25 (1.21-1.29)	<0.0001	1.38 (1.35-1.42)	<0.0001	1.27 (1.13-1.42)	<0.0001
4: Deprived areas, urban and rural, with poor overall health status of the population	1.48 (1.45-1.52)	<0.0001	1.35 (1.31-1.38)	<0.0001	1.13 (1.08-1.18)	<0.0001	1.41 (1.37-1.45)	<0.0001	1.42 (1.38-1.45)	<0.0001	1.62 (1.46-1.79)	<0.0001
5: Cities with abundant healthcare supply and heterogeneous	1.40 (1.37-1.43)	<0.0001	1.30 (1.27-1.33)	<0.0001	0.91 (0.87-0.95)	<0.0001	1.41 (1.37-1.45)	<0.0001	1.34 (1.31-1.37)	<0.0001	1.30 (1.18-1.44)	<0.0001

socio-economic situations												
6: Wealthy cities and sub-urban areas	1.10 (1.07-1.12)	<0.0001	1.04 (1.01-1.07)	0.0073	0.73 (0.69-0.76)	<0.0001	1.13 (1.10-1.16)	<0.0001	1.25 (1.22-1.28)	<0.0001	1.04 (0.94-1.16)	n.s.
Urban area zoning (ref: isolated municipality located outside the sphere of influence of a urban center)												
Large urban center	1.25 (1.22-1.28)	<0.0001	1.26 (1.23-1.29)	<0.0001	0.99 (0.95-1.03)	n.s.	1.35 (1.32-1.39)	<0.0001	1.03 (1.01-1.05)	0.0154	1.00 (0.91-1.10)	n.s.
Suburban municipality of a large urban center	1.11 (1.09-1.14)	<0.0001	1.10 (1.08-1.13)	<0.0001	0.91 (0.88-0.95)	<0.0001	1.16 (1.14-1.20)	<0.0001	1.01 (0.98-1.03)	n.s.	0.92 (0.83-1.02)	n.s.
Suburban municipality of several large urban centers	1.11 (1.08-1.14)	<0.0001	1.09 (1.07-1.12)	<0.0001	0.91 (0.87-0.96)	0.0001	1.15 (1.12-1.19)	<0.0001	1.00 (0.97-1.02)	n.s.	1.05 (0.94-1.18)	n.s.
Average urban center	1.40 (1.36-1.43)	<0.0001	1.40 (1.36-1.44)	<0.0001	1.02 (0.97-1.07)	n.s.	1.52 (1.48-1.57)	<0.0001	1.02 (1.00-1.05)	n.s.	1.11 (0.99-1.24)	n.s.
Suburban municipality of an average urban center	1.26 (1.19-1.33)	<0.0001	1.19 (1.13-1.25)	<0.0001	0.85 (0.77-0.94)	0.0012	1.30 (1.23-1.37)	<0.0001	0.96 (0.91-1.02)	n.s.	1.06 (0.84-1.35)	n.s.
Small urban center	1.10 (1.07-1.13)	<0.0001	1.12 (1.09-1.15)	<0.0001	0.95 (0.91-1.00)	0.0462	1.17 (1.14-1.21)	<0.0001	1.03 (1.00-1.05)	n.s.	1.11 (0.99-1.24)	n.s.

International Journal of
Environmental Research and Public Health

MDPI

Suburban municipality of a small urban center	1.16 (1.07-1.25)	0.0002	1.22 (1.13-1.32)	<0.0001	0.89 (0.77-1.02)	n.s.	1.32 (1.21-1.43)	<0.0001	1.05 (0.97-1.14)	n.s.	1.12 (0.81-1.56)	n.s.
Suburban municipality of several average or small urban centers	1.12 (1.09-1.14)	<0.0001	1.10 (1.07-1.13)	<0.0001	0.94 (0.90-0.99)	0.0098	1.15 (1.12-1.19)	<0.0001	0.99 (0.97-1.02)	n.s.	0.96 (0.86-1.07)	n.s.

[1] Excluding hospitalizations in somatic departments for psychiatric conditions or suicide attempts.

[2] Hospitalizations for asthma, congestive heart failure, chronic obstructive pulmonary disease, dehydration, complications of diabetes, angina (chest pain), dental problem, nutritional deficiency, conditions following immunization.
